# Energy harvesting performance of an EDLC power generator based on pure water and glycerol mixture: analytical modeling and experimental validation

**DOI:** 10.1038/s41598-021-02964-y

**Published:** 2021-12-06

**Authors:** Dong Kim, Dae Yeon Kim, Jaesool Shim, Kyung Chun Kim

**Affiliations:** 1 Rolls-Royce University Technology Center, Pusan National University, Busan, 46241 Republic of Korea; 2 School of Mechanical Engineering, Pusan National University, Busan, 46241 Republic of Korea; 3 School of Mechanical Engineering, Yeungnam University, Gyeongsan, 38541 Republic of Korea

**Keywords:** Energy science and technology, Energy harvesting

## Abstract

A liquid droplet oscillating between two plane electrodes was visualized, and the electrical power generation based on the reverse-electrowetting-on-dielectric (REWOD) phenomenon was measured. For the upper plate, a hydrophobic surface treated by PTFE was used, and the lower plate was tested using the hydrophilic surface properties of ITO glass. To analyze the dynamic behavior of an oscillating liquid bridge, a modeling study was carried out using the phase field method based on the finite element method. The dynamic contact angle of the oscillating liquid bridge was modeled based on advancing and receding contact angles. The variable interfacial areas between the liquid and solid surfaces were calculated and agreed well with the experimental results within a 10% error band. Furthermore, experimental and analytical studies were carried out to examine the REWOD energy harvesting characteristics of the glycerol-water mixtures in various concentrations. As a result, the peak voltage output was obtained at a specific concentration of the glycerol mixture, and the power density of the oscillating liquid bridge at this point was up to 2.23 times higher than that of pure water.

## Introduction

Energy harvesting is undoubtedly a very attractive technique for a wide variety of self-powered microsystems, such as wireless sensors, biomedical implants, machinery monitoring devices, structure-embedded instrumentation, remote weather station, and wearable or portable electronic devices. Over the past decade, the number of research reports has continued to increase as interest in energy harvesting has increased^1–3^. Piezoelectric, electrostatic, and electromagnetic generators are well known as conventional energy-harvesting techniques, and research into those methods has been widely conducted^3–8^. Recently, the reverse electrowetting on dielectric (REWOD) method has emerged as a novel energy-harvesting technique that uses the reverse process of the electrowetting on dielectric (EWOD) method. This technology has several advantages compared to conventional energy-harvesting methods.

Conventional mechanical energy-harvesting methods produce an appropriate output when energy is generated by a strong force or a high frequency. However, environmental energy from sources such as transportation and human motion is generated by weak forces at low frequencies. Compared with conventional methods, REWOD tends to produce a relatively high output density. Thus, it is suitable for use with low power and low frequencies. In addition, a wide range of power can be produced without using a separate system^9–11^. The use of miniaturized and permanently available energy harvesters instead of batteries could improve environmental problems, and REWOD energy-harvesting technology is of great interest in academia and industry^4,12,13^.

Krupenkin and Taylor^9^ suggested a new approach for generating electrical energy in micro-fluidic systems from vibrational environment using reverse electro wetting. They reported a high-efficiency energy-harvesting device by combining REWOD phenomena with a high-frequency self-oscillation process that occurs during the growth and collapse of an air bubble^10^. Yang et al.^11^ deposited an Al_2_O_3_ film with a higher dielectric constant by atomic layer deposition (ALD) to improve the efficiency of the device. The leakage current density decreased significantly in the case of ALD films in comparison to a sputtered film. They improved the device performance by reducing the DC bias voltage to 24 V and the external frequency of vibration to 2 Hz. In contrast, Krupenkin and Taylor’s method used a frequency of up to 300 Hz was used.

However, in this technique, the liquid metals used as working fluids (Galinstan and mercury) are toxic. Janssen et al.^14^ further studied the concept thermodynamically and mechanically and extended it to any type of liquid bridges between oscillating electrodes. Huynh et al.^15^ proposed an electrostatic micro-power generator where the change in capacitance is achieved by the sliding movement of an aqueous solution of NaCl. However, an external DC bias-voltage source is still required.

Moon et al.^16^ proposed a new method for electrical power generation using pure water without electromagnetic induction. The electrical double layers were mechanically modulated to increase the suitability for practical applications. Kwon et al.^17^ proposed an energy-harvesting method with the sliding of water droplets and no external bias voltage sources. This was extended to ionic liquids as the working fluid with a wide operating temperature of up to 100 °C to enhance the power generation method by Kong et al.^18^. Wu et al.^13^ proposed a hydrogel-based energy-harvesting method for common broad bandwidth vibration sources (0–80 Hz).

To carry out parametric studies on REWOD, a fundamental analysis of the dynamic behavior of an oscillating liquid bridge is essential. Liquid bridges formed between two solid surfaces are commonly observed in nature and have been studied for their importance in industrial applications. The stability of liquid bridges subject to shear-induced flow was characterized by Uguz et al.^19^ and the stability of static liquid bridge between nonparallel hydrophilic surfaces was experimentally and numerically studied by Ataei et al.^20^. However, most of those studies focused on static or quasi-static motion of a liquid bridge between two solid surfaces.

In this study, the dynamic behavior of a vibrating droplet column between hydrophobic and hydrophilic surfaces was modeled. The liquid deformation model was combined with a resistor–capacitor (RC) circuit model to calculate the electrical power generation from the electrical double layer capacitors (EDLC), and the numerical results were validated with the experimental results. Furthermore, the effect of the mixture of glycerol and pure water was investigated along with how the harvesting efficiency of an oscillating liquid bridge is improved by modulating the glycerol concentration that easily control the physical properties of the working fluids.

## Experimental setup

**Figure 1:**
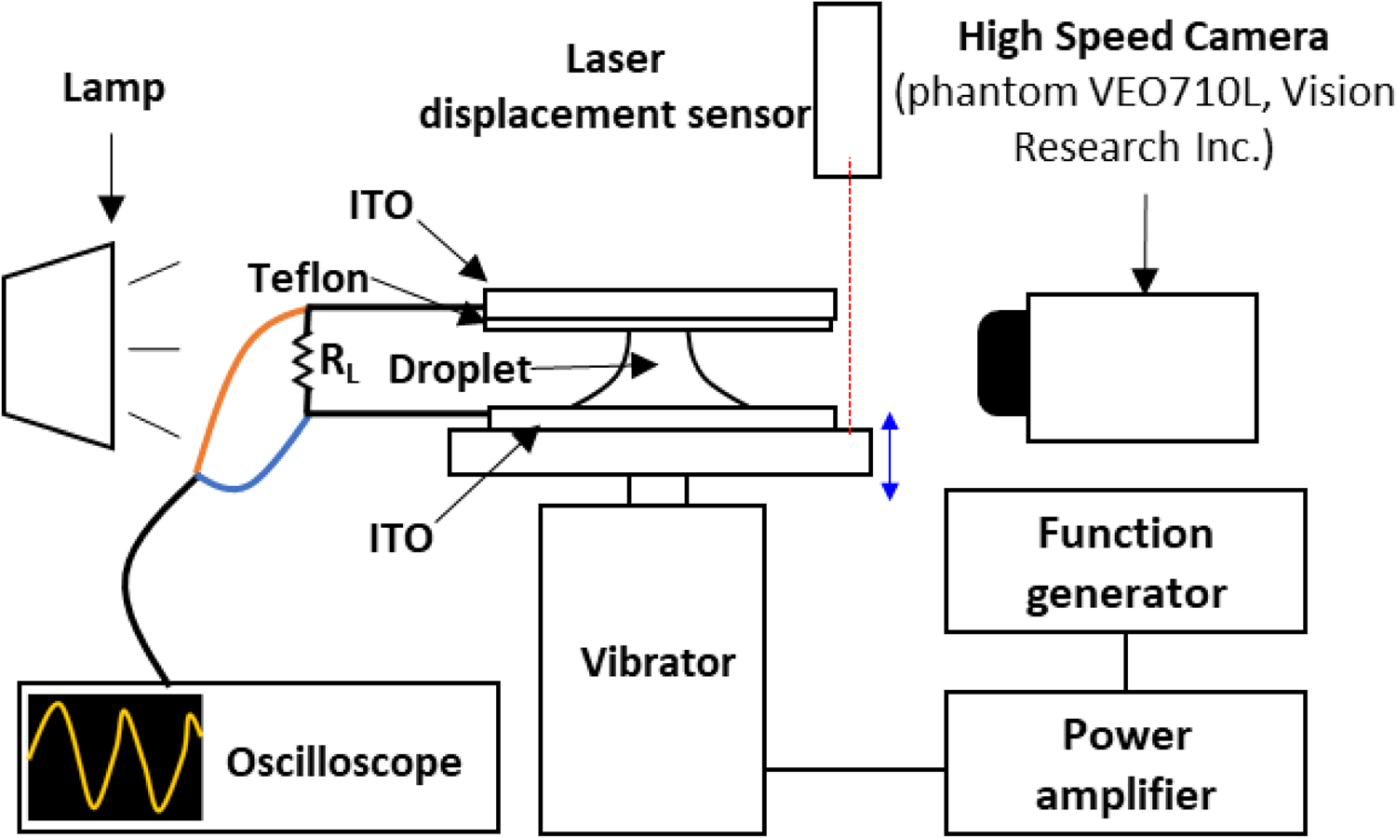
Diagram of experimental setup.

A diagram of the experimental setup is shown in Fig. 1. A liquid bridge made of deionized water is formed between two parallel indium tin oxide (ITO)-coated glass plates. The upper side of the ITO glass was coated with hydrophobic polytetrafluoroethylene (PTFE). Teflon-amorphous fluoropolymer (AF) was diluted in a fluorocarbon solvent of FC-40 at a concentration of 0.6 wt%, and the Teflon solution was dip-coated onto the ITO glass. The samples were then baked at 200 °C for 30 min. The contact angle of the hydrophilic ITO glass surface was 62.5°, and that of the hydrophobic PTFE-coated surface was 107°. Also, the thickness of the dielectric layer that was coated by PTFE on the top ITO glass was measured by Alpha-Step and SEM and the thickness of the layer is about 300 nm.

A sine-wave signal was generated by a function generator (33250A, Agilent), amplified by a power amplifier (EA200S, Elizer), and transmitted to a vibrator (ET-132-2, Labworks) to shake the bottom plate. An oscilloscope (TDS2024C, Tektronix) measured the voltage drops at the load resistance (10 MΩ). A laser displacement sensor (HL-G1, Panasonic) measured the amplitude of the vibrator. A high-speed camera (phantom VEO710L) took photos at frame rates of 1000 and 3000 fps. The contact line and contact angle were measured by image processing with MATLAB.

**Figure 2:**
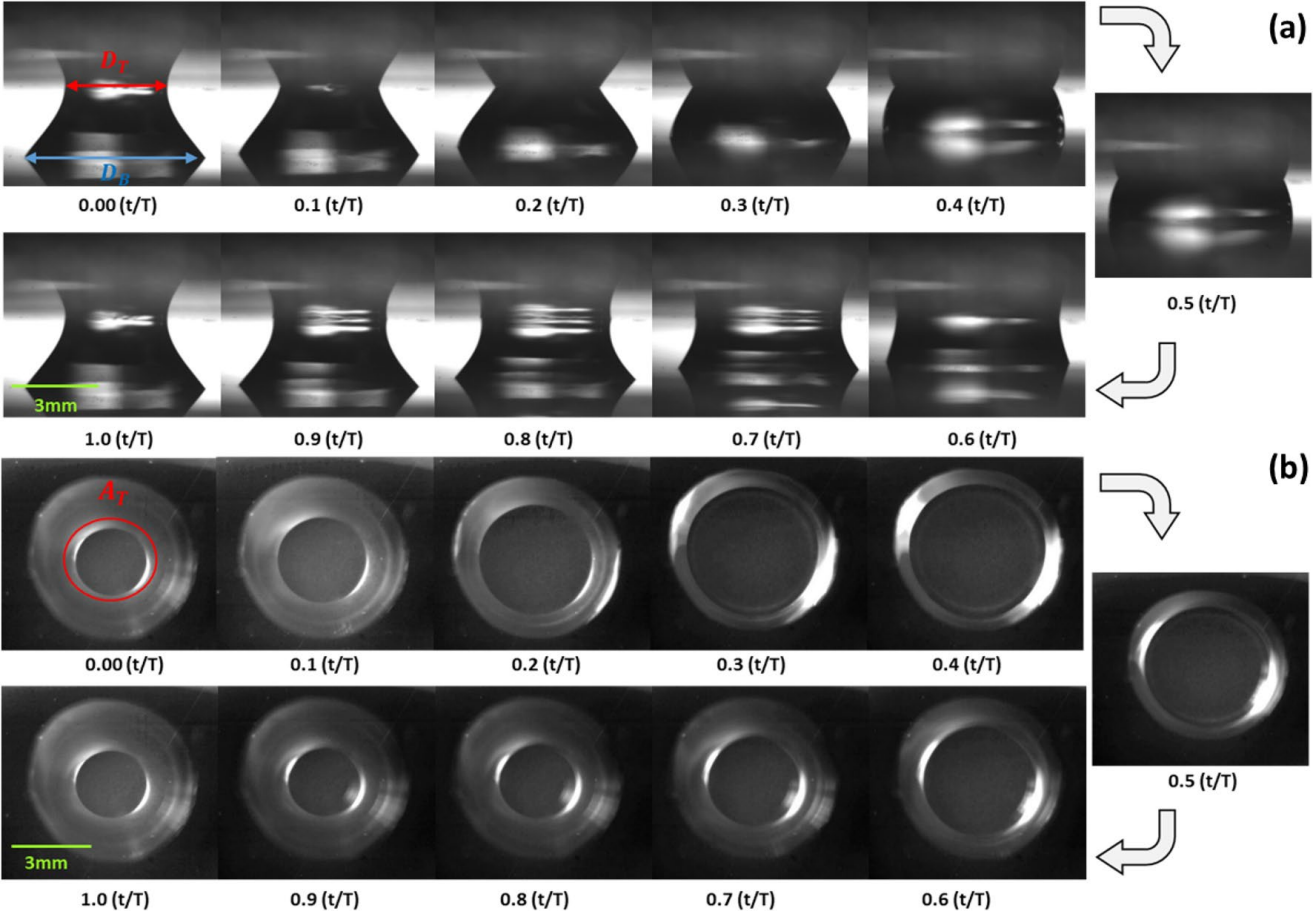
(**a**) Front view and (**b**) top view of an oscillating liquid bridge between hydrohpobic (top side) and hydrophilic (bottom side) surfaces at a frequency of 10 Hz and vibration amplitude of 0.50 mm.

**Figure 3:**
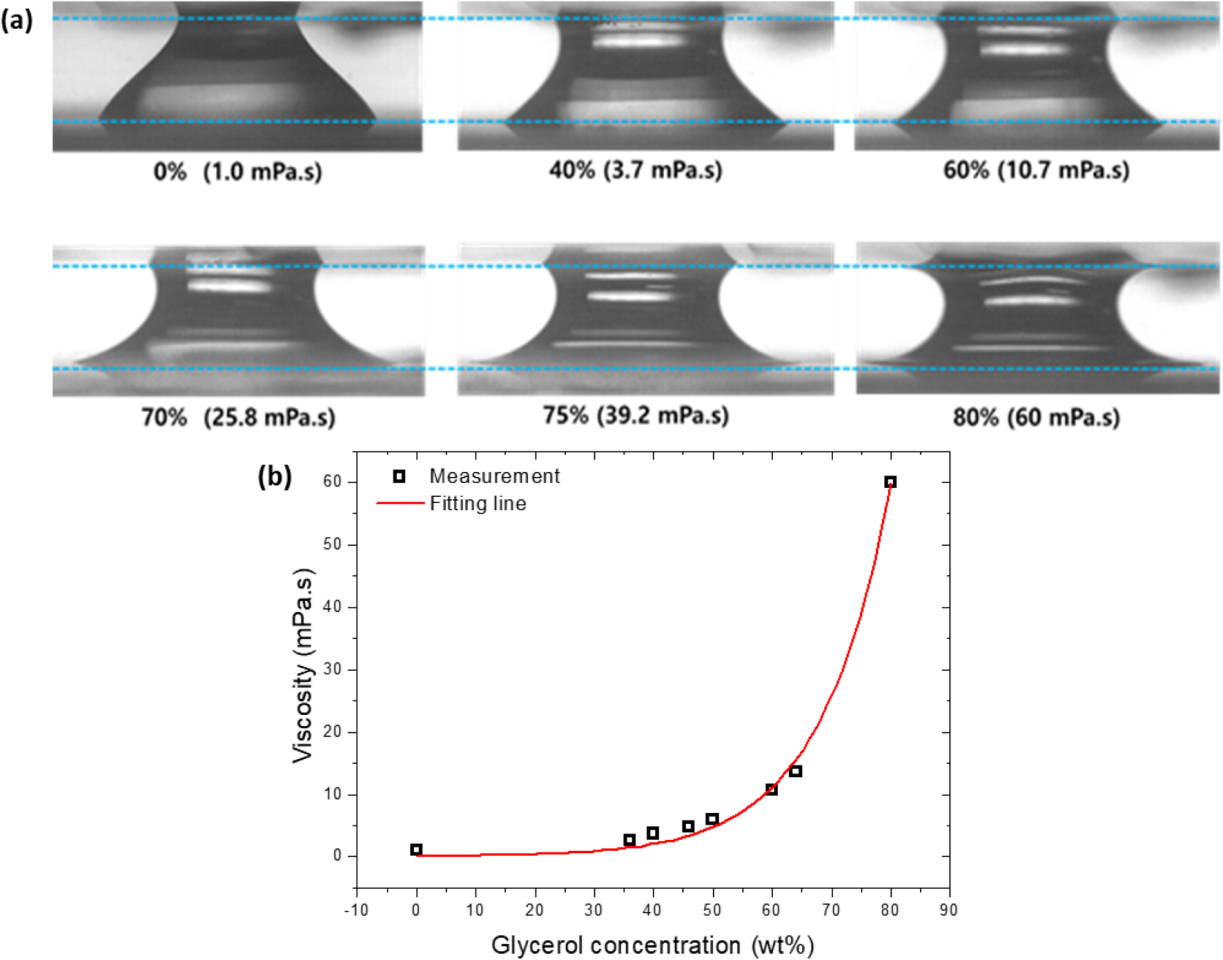
(**a**) Different shapes of liquid bridges and (**b**) the viscosity according to various concentrations of the glycerol mixture.

Images taken from the front side and top side over time are shown in Fig. 2^21^. The bottom glass is hydrophilic, while the top surface is hydrophobic. As a result, the bottom contact area is stable, but the top area periodically changes during the oscillation. There is a difference between the top contact area and the bottom contact area because of the gravity effect and the lower capillarity of the top plate. In the oscillating process, the bottom contact area keeps the initial state all the time because of the pinning effect caused by the hydrophilicity of bottom plate. On the other hand, the top contact area changes periodically as the bottom plate moves up and down because the top plate was hydrophobically coated with PTFE. The size of a liquid drop is 40 μl, the vibration frequency is 10 Hz, and the amplitudes are 0.50, 0.75 and 0.90 mm. To investigate the effect of the glycerol mixture when it used as a working fluid, experiments were done using various concentrations of the glycerol-water mixture of 0 to 80% by weight. Figure 3 shows the viscosity of the glycerol mixture. The viscosity was up to 60 mPa s. As the concentration increases, the liquid bridge attaches more to the plates, and the contact angle decreases as shown in Fig. 3a.

## Numerical model

### Modeling of liquid deformations

Eulerian approaches for handling the mobbing boundaries have been reported for interface tracking by a fixed Eulerian grid using a scalar indicator function^22,23^, which are classified as the volume-of-fluid (VOF) method, the level set method, and the phase field method. The phase field method is one of the most attractive tools that can deal with interfacial problems, formulate the true-to-motion behavior of complex interfaces, and treat the topological changes of the interface between two fluids^24^.

**Figure 4:**
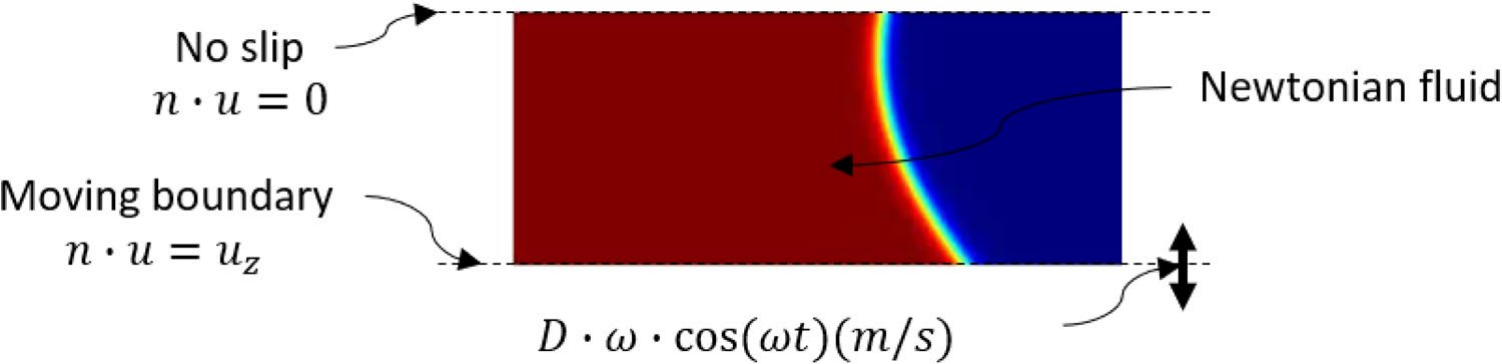
Boundary conditions and assumptions for the liquid deformation model.

Figure 4 shows the boundary conditions and assumptions for the liquid deformation model from front view. Red and blue colors show the working fluids and air, respectively. The initial surface distance (height) was set to 1.7 mm. For incompressible fluids, such as a liquid droplet, the Navier–Stokes equation including a gravity force term $F_{g}$ having the value of $\rho$ g and surface tension force term $F_{\sigma }$ is employed.

1 $$\rho \left( {\frac{\partial u}{{\partial t}} + u \cdot \nabla u} \right) = \nabla \cdot \left( { - p + \eta \nabla u} \right) + F_{g} + F_{\sigma }$$

2 $$\nabla \cdot u = 0$$

3 $$F_{\sigma } = \lambda \left[ { - \nabla^{2} \varphi + \frac{{\varphi \left( {\varphi^{2} - 1} \right)}}{{\varepsilon^{2} }}} \right]\nabla \varphi$$

*λ* is the mobility parameter, which determines the time scale of the Cahn–Hilard diffusion, while *ε* is the interface thickness-controlling parameter, and *φ* is the shape of the interface. The fluid-air interface can be described by the phase-field method.

4 $$\lambda = \frac{3\sigma \varepsilon }{{2\sqrt 2 }}$$

5 $$\frac{\partial \varphi }{{\partial t}} + u \cdot \nabla \varphi = \gamma \lambda \nabla^{2} \left[ { - \nabla^{2} \varphi + \frac{{\varphi \left( {\varphi^{2} - 1} \right)}}{{\varepsilon^{2} }}} \right]$$

6 $$\rho = \rho_{liquid} + \left( {\rho_{gas} - \rho_{liquid} } \right)\left( {\frac{1 + \varphi }{2}} \right)$$

7 $$\mu = \mu_{liquid} + \left( {\mu_{gas} - \mu_{liquid} } \right)\left( {\frac{1 + \varphi }{2}} \right)$$

8 $$\varphi = \left\{ {\begin{array}{*{20}l} { - 1 < \varphi < 0} \hfill & {at\;gas\;region} \hfill \\ 0 \hfill & {at\; interface} \hfill \\ {0 < \varphi < 1} \hfill & {at\;liquid\;region} \hfill \\ \end{array} } \right\}$$

$\rho$ is density, $\mu$ is dynamic viscosity of fluid and γ is the surface tension. The bottom plate having length D = 4 mm was assumed to move in only the z-direction with sinusoidal velocity.

9 $$u_{z} \left( t \right) = D \cdot \omega \cdot \cos (\omega t)\quad \left[ {{\text{m}}/{\text{s}}} \right]$$

The upper plate had no slip conditions, and the lower plate had moving boundary conditions.

10 $$n \cdot u = 0; n \cdot u = u_{z}$$

The fluid is Newtonian, and no contact occurs between the two plates.

The contact angle $\left( \alpha \right)$ is described with respect to both the upper and lower boundaries as follows:

11 $$n \cdot \varepsilon^{2} \nabla \varphi = \varepsilon^{2} \tan \left( {\frac{\pi }{2} - \alpha } \right)\left| {\nabla \varphi - \left( {n \cdot \nabla \varphi } \right)n} \right|$$

12 $$n \cdot \left( {\frac{\gamma \lambda }{{\varepsilon^{2} }}} \right)\nabla \left( { - \nabla \cdot \varepsilon^{2} \nabla \varphi + \varphi \left( {\varphi^{2} - 1} \right)} \right) = 0$$

The commercial code COMSOL Multiphysics was used, which is based on FEM. The governing equations were discretized using the Galerkin formulation. LU factorization (unsymmetric-pattern multi-frontal numerical factorization) was used for the solver. Convergence criteria were set for the momentum and the fluid-air interface equation for the phase field method, respectively. Here the subscripts n and n-1 denote the current and the previous iteration step, respectively.

13 $$\left| {\frac{{u_{n} - u_{n - 1} }}{{u_{n} }}} \right| \le 10^{ - 5} ; \left| {\frac{{\varphi_{n} - \varphi_{n - 1} }}{{\varphi_{n} }}} \right| \le 10^{ - 5}$$

### Modeling of RC circuit with EDLC (electrical double layer capacitors)

**Figure 5:**
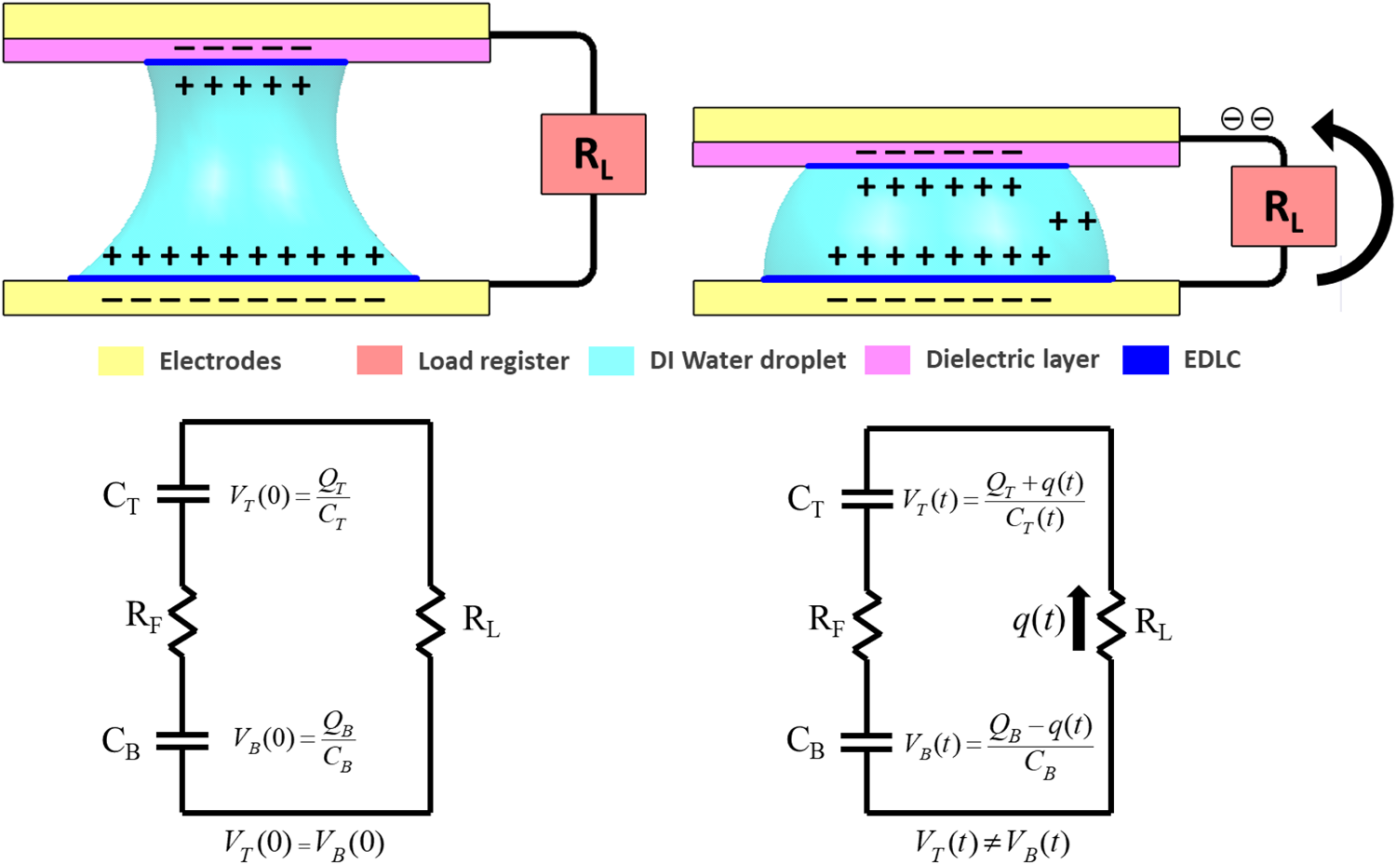
Schematics of RC-circuit model with EDLC.

Figure 5 shows the procedure of energy harvesting from the oscillating liquid bridge between two parallel electrodes. When a dielectric fluid contacts a conductive solid surface, electric charges are created on the interfacial surfaces and attract counter ions from the liquid, which has an exponentially decreasing distribution from near the surface. This system is called an electrical double layer^25,26^ or an electrical double layer capacitor because its structure is similar to that of an electric capacitor^27,28^.

Two different EDLCs on the top and bottom sides between the liquid and electrode surfaces are created in the circuit. It is assumed that both side capacitances of the EDLCs, the capacity of top electrode, *C*_*T*_ and that of bottom electrode, *C*_*B*_, increase linearly with the change rate of the top contact areas *A*_*T*_ and the bottom contact area *A*_*B*_*,* as in the following relations^29^:

14 $$C_{T} \left( t \right) = \varepsilon_{o} A_{T} \left( t \right)\left( {\frac{d}{{\varepsilon_{P} }} + \frac{{\lambda_{D} }}{{\varepsilon_{d} }}} \right)^{ - 1} \cong \frac{{\varepsilon_{o} \varepsilon_{p} }}{d}A_{T} \left( t \right); C_{B} \left( t \right) \cong \frac{{\varepsilon_{o} \varepsilon_{d} }}{{\lambda_{D} }}A_{B} \left( t \right) \cong Const.$$

where *d* is the thickness of the PTFE coating layer on the ITO surface of the top plate, λ_D_ is the characteristic thickness of the bottom EDL, ε_*o*_ is the dielectric constant in a vacuum, ε_*p*_ is the dielectric constant of the PTFE layer, and ε_*d*_ is the dielectric constant of a droplet, as shown in Table 1. These parameters are applied in the calculation process. In this study, *d/*ε_*p*_ is much higher than λ_D_/ε_*d*_, so the approximation in Eq. (14) is reasonable^16,29,30^. Since the change of the bottom contact area is relatively negligible compared to the upper side, the capacitance of the bottom EDLC is approximated to a constant value.

**Table 1:** Input parameters for EDLC model of pure water.

Parameter	ε_0_ (F/m)	ε_p_ (-)	ε_d_ (-)	d (m)	λ_D_ (m)	R_F_ (MΩ)	R_L_ (MΩ)
Value	8.85E−12	2.1	78	300E−9	300E−9	2	10

In the equilibrium state, no current flows between two EDLCs, so the system has zero potential. However, when the oscillation starts, the EDLCs are periodically charged and discharged due to the continuous change of the top contact area between the liquid and the ITO surface, and electrical current is generated due to the periodic imbalance between the two EDLCs. This procedure demonstrates the RC-circuit model to calculate the voltage generation through the oscillating liquid bridge. This circuit can be characterized by the following differential equation:

15 $$\left( {R_{F} + R_{L} } \right)\frac{{dq\left( {t_{n} } \right)}}{dt} \equiv \Delta V_{B - T} \left( {t_{n} } \right) = \frac{{Q_{B} \left( {t_{n - 1} } \right) - q\left( {t_{n} } \right)}}{{C_{B} }} - \frac{{Q_{T} \left( {t_{n - 1} } \right) + q\left( {t_{n} } \right)}}{{C_{T} \left( {t_{n} } \right)}}; q\left( {t_{1} } \right) = 0$$

where R_F_ means the resistor of the fluid, R_L_ means the load resistor and $\Delta V_{B - T} \left( t \right)$ is the voltage drop between the bottom and top interface. The *q*(*t*) is the change in charge in the top or bottom EDLC at time *t* flowing through the load register between the two electrodes. If the induced oscillation at the previous time step changes the top contact area, then the capacity of the top EDLC changes. The potential charge can be obtained from Eq. (15) and generates an AC electrical current *dq*/*dt*. The voltage drop on *R*_L_ is as follows:

16 $$V_{L} \left( t \right) = V\left( t \right)R_{L} /\left( {R_{F} + R_{L} } \right)$$

## Results and discussion

### Dynamic contact angle

**Figure 6:**
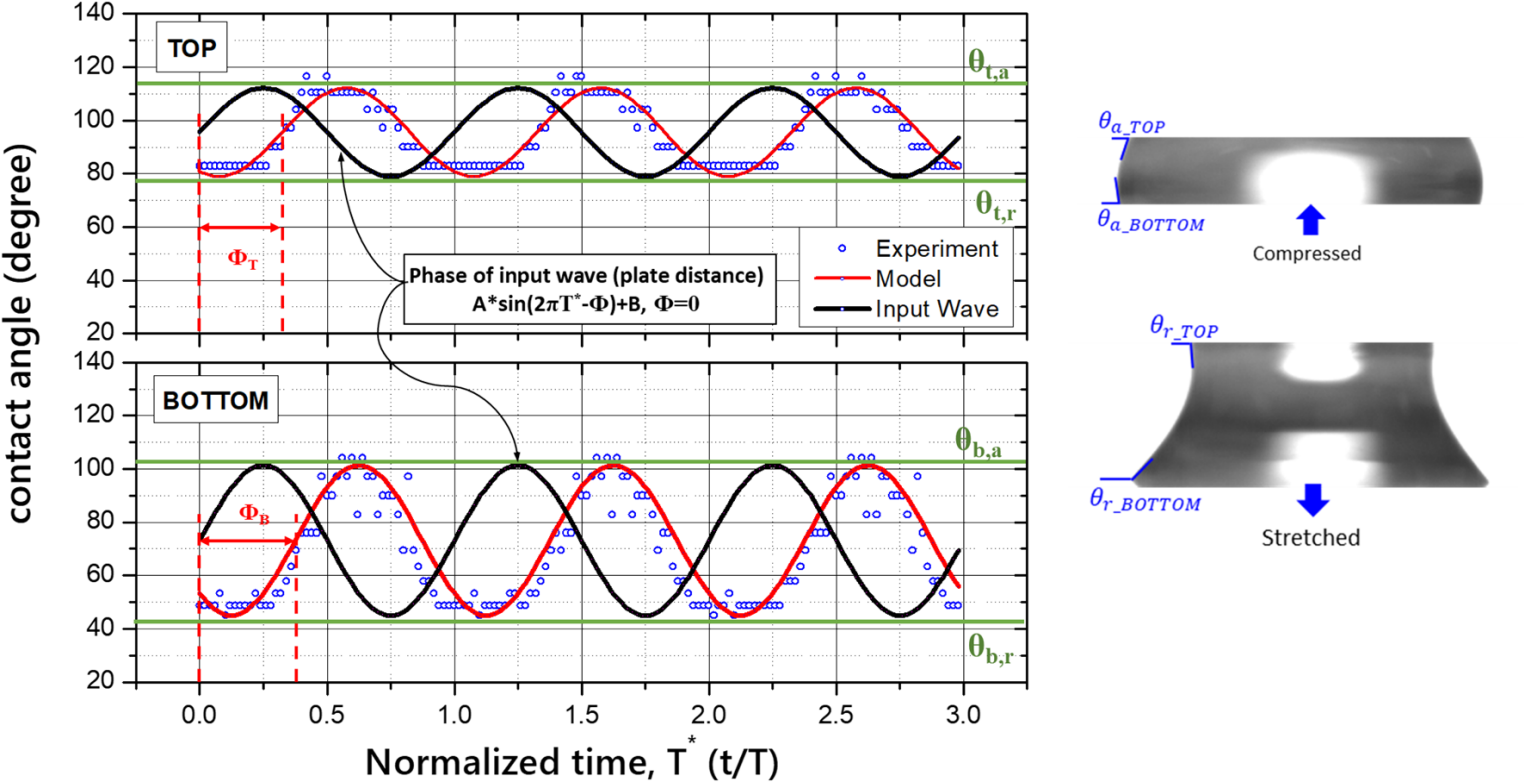
Measured contact angle on the top and bottom sides from the experiment according to the normalized time T* and comparison with input oscillatory signal.

Figure 6 shows the contact angles between the top and bottom contacting surfaces and the curvature of a pure water drop. The angles were captured from the experimental images according to the normalized time T^*^(= t/T) and compared with the zero-phase input sine wave of the shaker, which is indicated by a black line. The advanced contact angle θ_a_ is the maximum contact angle when the liquid bridge is compressed, and the receding contact angle θ_r_ is the minimum contact angle when the bridge is stretched. The phase of the contact angle was shifted from the input vibration wave as + 0.65π on the top side and + 0.75π on the bottom side. The phase shift between the top and bottom contact angles is π /10. Therefore, the dynamic contact angle can be simply modeled using the measured advancing contact angle θ_a_ and receding contact angle θ_r_:

17 $$\begin{aligned} CA_{top} & = \frac{{\left( {\theta_{t,a} - \theta_{t,r} } \right)\sin \left( {\frac{2\pi t}{T} - \Phi_{t} } \right)}}{2} + \frac{{\left( {\theta_{t,a} + \theta_{t,r} } \right)}}{2}; \\ CA_{bottom} & = \frac{{\left( {\theta_{b,a} - \theta_{b,r} } \right)\sin \left( {\frac{2\pi t}{T} - \Phi_{b} } \right)}}{2} + \frac{{\left( {\theta_{b,a} + \theta_{b,r} } \right)}}{2} \\ \end{aligned}$$

The phase shift of the top side, Φ_t_, is 0.65π, and that of the bottom side, Φ_b_, is 0.75π. The overall error of this model is 12.5%.

### Model validations

**Figure 7:**
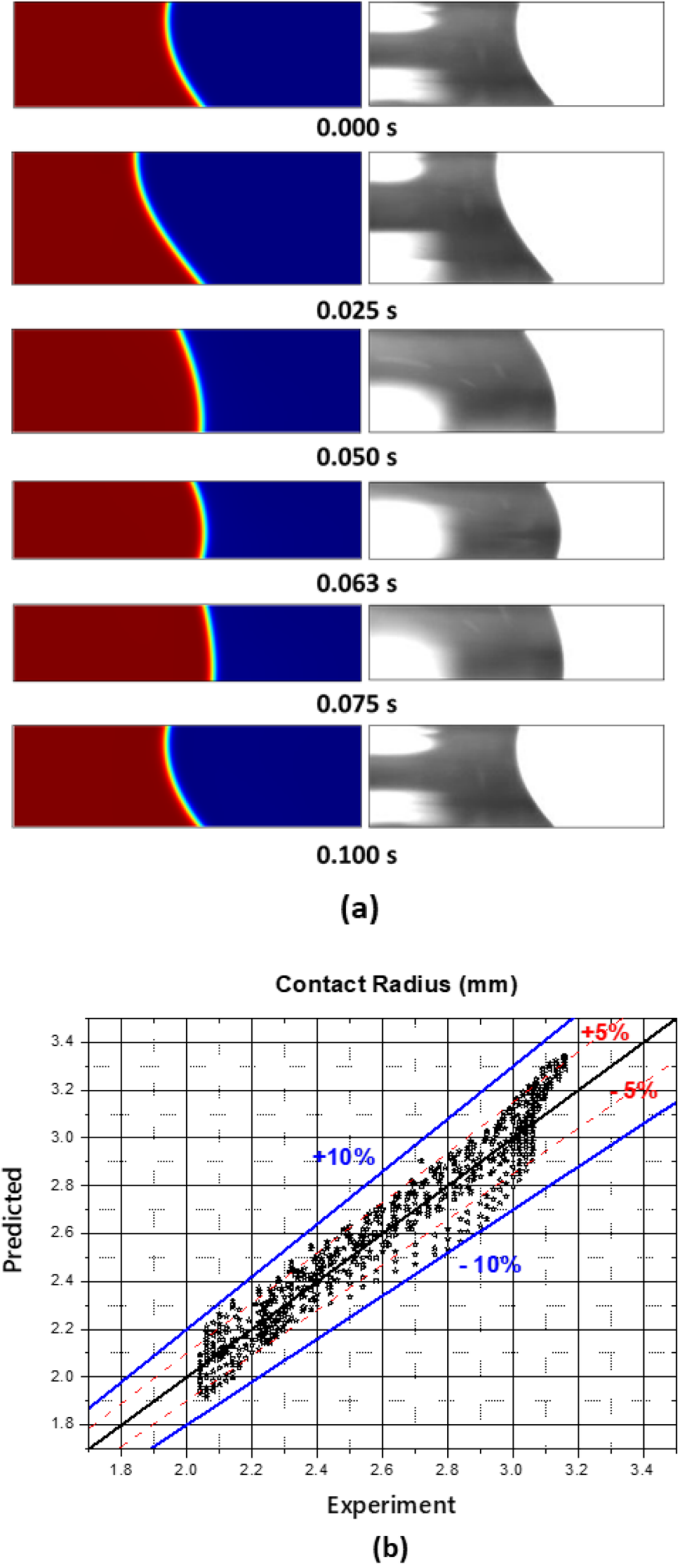
Comparison between the experimental result and the simulation. (**a**) Liquid deformations and (**b**) contact radius.

The calculated contact radius of the oscillating liquid bridge was compared with experimental images for each time step, as shown in Fig. 7a. A drop of 40 μl of pure DI water was used at 10 Hz with a vibration amplitude of 0.50 mm. Figure 7b shows 1,061 data points in comparison with the experimental data during three periods of the induced oscillation. The results agreed well with the experimental data and mostly fell within a 5% error range.

**Figure 8:**
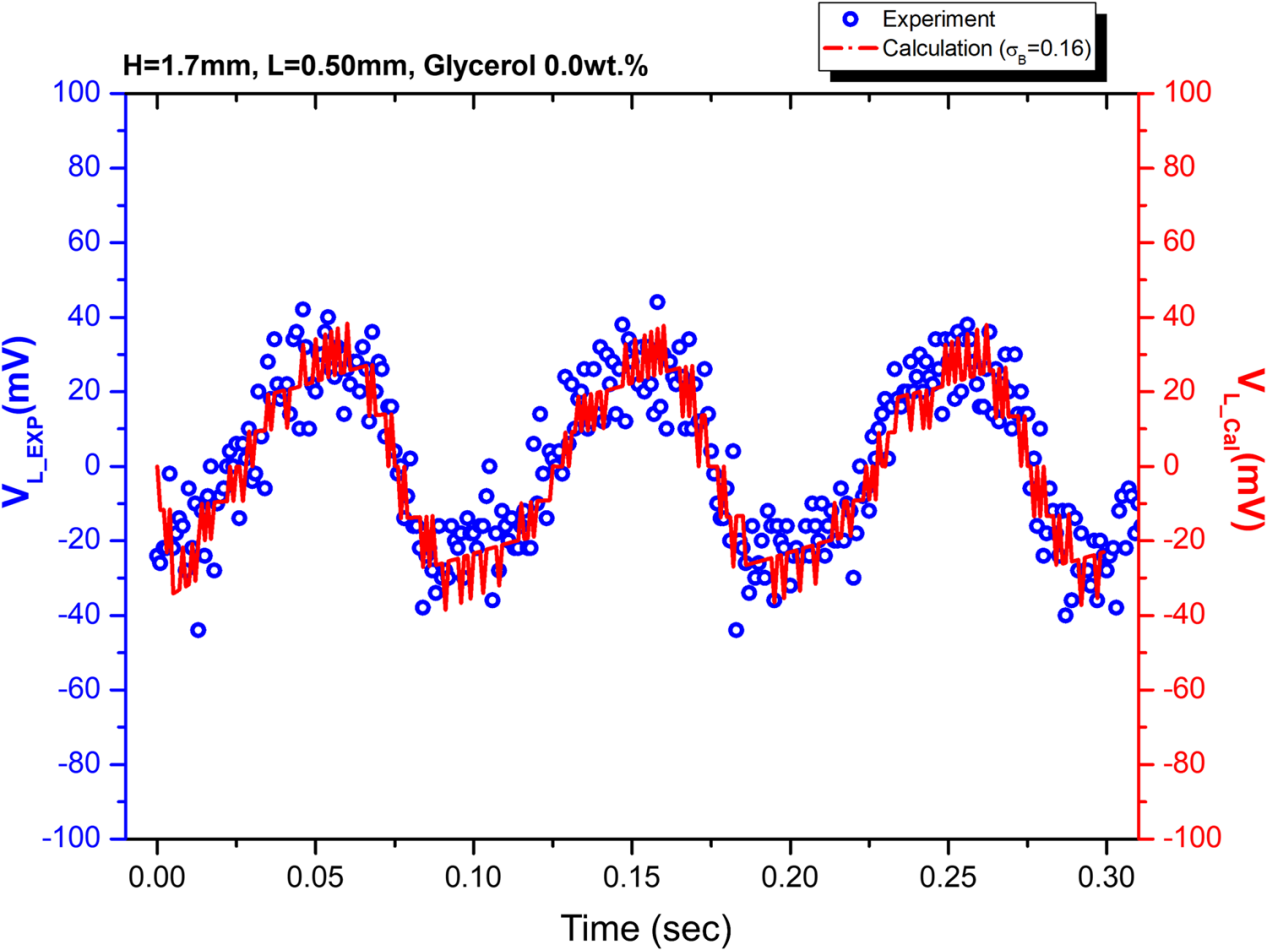
Experiment (blue circles) and numerical calculations (red curves) of voltage drop V_L_(t) through load resistor R_L_ as a function of time with sinusoidal wave inputs at 10 Hz with a different amplitude of 0.50 mm.

The voltage output of the oscillating liquid bridges was calculated using Eq. (16) and compared with the experimental results in Fig. 8. The calculation agrees well with the experimental results.

### Effects of glycerol mixture

**Figure 9:**
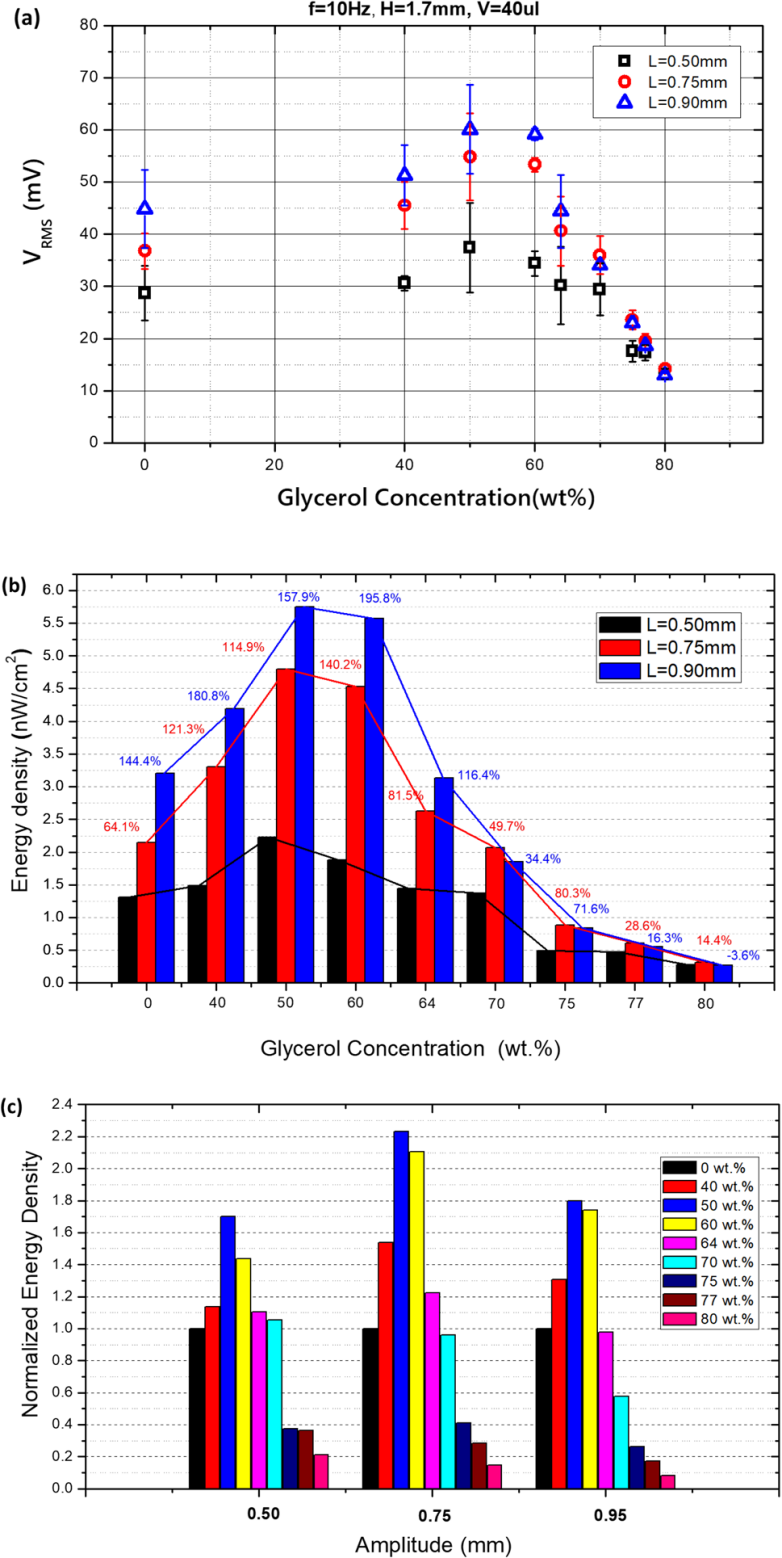
Experimental results of energy output: (**a**) V_RMS_, (**b**) energy density, and (**c**) normalized energy density based on pure water.

Fig. 9a shows the experimental results of the generated voltage according to the concentration of glycerol. As the amplitude of the vibration increases, the output RMS voltage also increases and becomes clearer. Interestingly, the peak output values are found at specific concentration points, especially in the region of glycerol concentration between 50 and 60 wt%.

Figure 9b shows the energy density (the electrical power generation divided by unit interfacial area) at different glycerol concentrations and vibration amplitudes. The indicated values show increases along with the vibrational amplitude compared to the lowest amplitude (L = 0.5 mm) for each concentration. As expected, the peak point of the energy density was observed at every amplitude. The maximum output of around 5.75 nW/cm^2^ was observed at an amplitude of L = 0.90 mm at a glycerol concentration of 50 wt%. However, the highest increment of 195.8% with respect to the amplitude was found at 60 wt%. When the concentration is higher than 70 wt%, the maximum energy density was observed at L = 0.75 mm, and when the amplitude increased, the output decreased. At a concentration of 80 wt%, the energy density decreased by – 3.6% compared to the case of L = 0.5 mm.

Figure 9c shows the normalized energy density (NED) based on the case of pure water in comparison to different glycerol concentrations for each amplitude of induced vibration. As the concentration increased, the maximum NED was 2.23 times that obtained at a concentration of 50 wt%. The NED decreased significantly at a concentration of 70 wt%, and at higher amplitudes, NEDs less than 1 can be found at lower concentrations.

**Figure 10:**
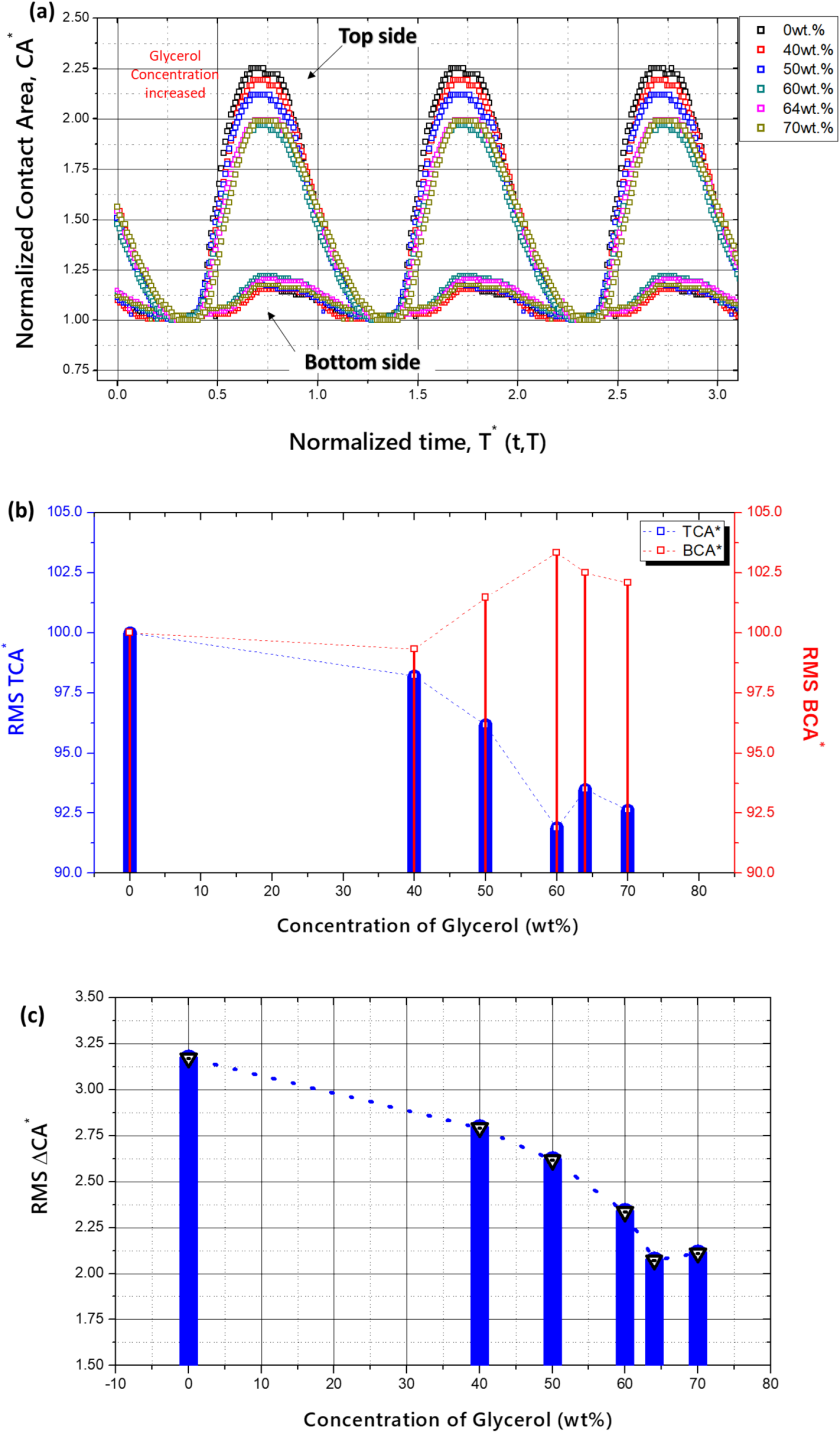
Contact area according to the glycerol concentration. (**a**) Normalized contact area, (**b**) root mean squared normalized contact area, and (**c**) differential contact area between bottom and top sides.

To find the reason why this peak point of the output voltage appears to be associated with the effect of the glycerol mixture, the contact areas on the top side (TCA) and bottom side (BCA) were normalized as TCA^*^ (= A_T_/A_T_min_) and BCA^*^ (= A_B_/A_B_min_), where A_min_ is the minimum contact area for each concentration of the glycerol mixture, and the results are compared in Fig. 10a. As a result, the CA^*^ values changed with the glycerol concentrations. However, the phase of the waves is constant when the vibration conditions are the same.

Fig. 10b shows the root mean squared values of the normalized top and bottom contact areas TCA^*^_RMS_, BCA^*^_RMS_. TCA^*^_RMS_ decreased, while the bottom side had a peak point, but when comparing both sides, the variation of BCA^*^_RMS_ was relatively small. The difference in contact area between the top and bottom sides was also normalized as ΔCA^*^(= ΔCA/ΔCA_min_), and the root mean squared values are shown in Fig. 10c. ΔCA^*^ decreased as the concentration of glycerol increased up to 34.6% compared to that of pure water. This can be explained based on Eqs. (14–17), which show that a higher concentration of glycerol leads to a lower capacity of the EDLC.

**Figure 11:**
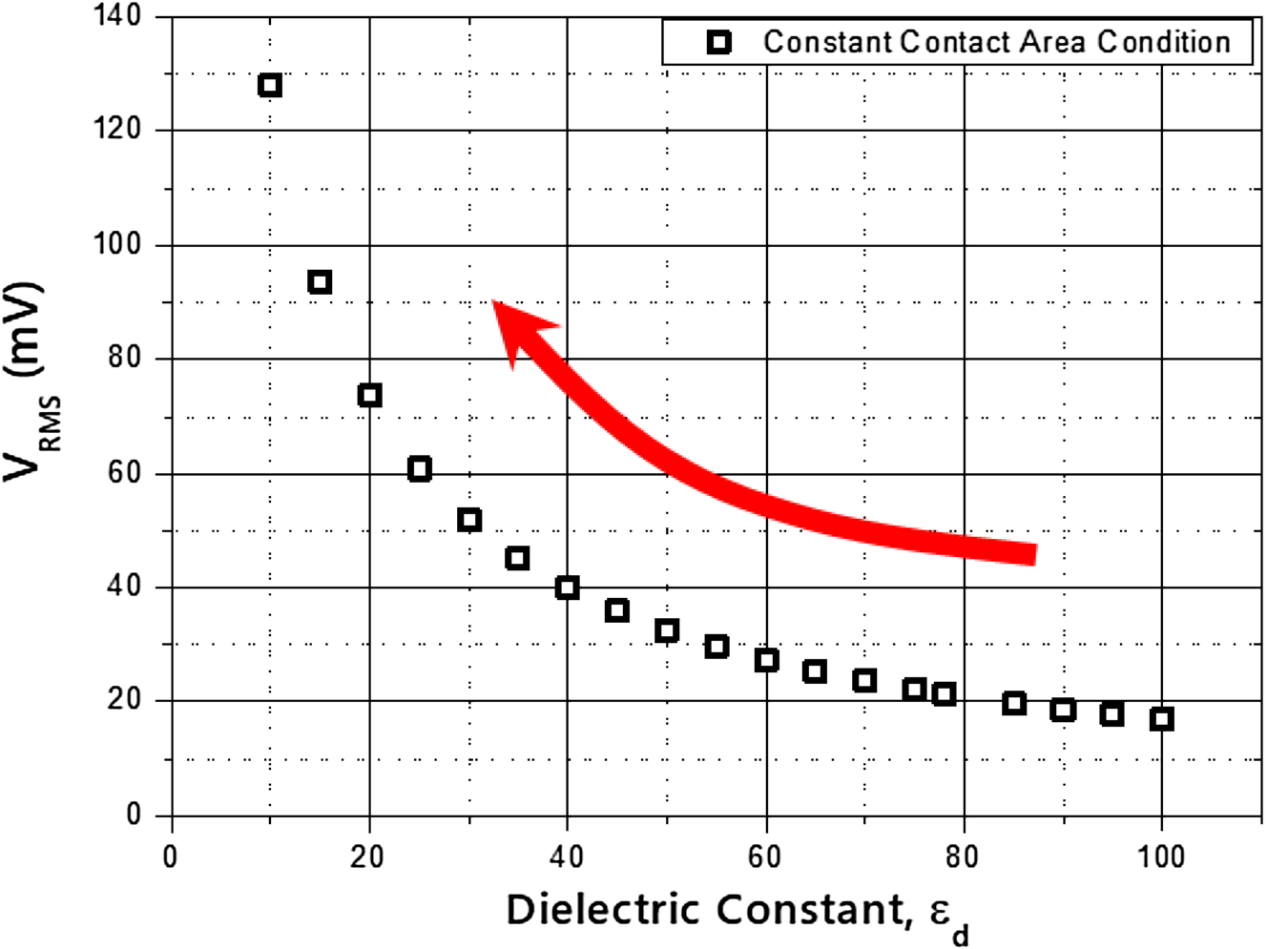
Relation between the dielectric constant and RMS voltage output calculated from the RC-circuit model.

However, considering the physical properties of the fluid, a greater glycerol concentration leads to a lower dielectric constant, as shown in Table 2, as well as lower viscosity, surface tension, and density of the mixture^31–33^. As the dielectric constant decreases, the output voltage increases, as shown in Fig. 11. The trade-off between the dynamic motion of the liquid bridge and the fluid properties produced the peak point in the output voltage at various concentrations of the glycerol mixture.

**Table 2:** Physical properties of the working fluids.

Glycerol concentration (wt%)	Viscosity μ (mPa s)*	Surface tension γ (dyne/cm)*	Density ρ (g/cm3)*	Dielectric constant ε (-)**
0	1.0	71.2	1.000	**78.0**
40	3.7	65.9	1.100	**67.1**
50	6.0	65.2	1.130	**65.1**
60	10.7	64.8	1.160	**62.0**
64	13.7	64.6	1.168	**60.0**
70	22.5	64.5	1.184	**59.1**

*Hong et al.^29,30^, **Glycerine Producers' Association. “Physical properties of glycerine and its solutions.” Glycerine Producers' Association, 1963^31^.

Significant values are in [bold].

**Figure 12:**
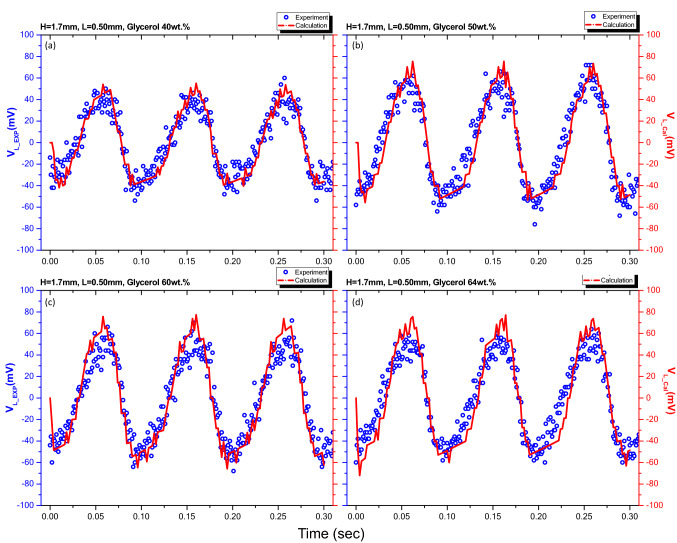
Comparison of the experimental results (blue circles) and predictive value (red line) of output voltage V_L_ at 10 Hz, 0.5 mm amplitude, and 1.7 mm initial height with various glycerol concentrations. (**a**) 40 wt%, (**b**) 50 wt%, (**c**) 60 wt%, (**d**) 64 wt%.

The voltage output of the oscillating liquid bridges was calculated for various concentrations of the glycerol mixture at a frequency of 10 Hz, initial height of 1.7 mm, and amplitude of 0.5 mm. The calculated results are compared with the experimental results in Fig. 12. As shown in the figure, the prediction agrees well with the experimental results.

**Figure 13:**
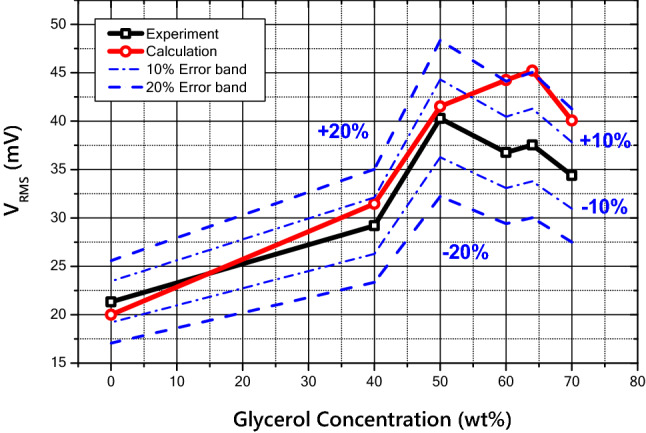
Comparison of RMS output voltage between experimental results and predictive model.

Finally, the root mean squared voltage output was predicted as a function of the glycerol concentration of the mixture and compared with the experimental results, as shown in Fig. 13. The predicted contact radius was calculated from the numerical analysis of the liquid deformation model, and the predicted voltage output was calculated using the RC-circuit model based on the predicted contact radius. As shown in the figure, the physical properties of the fluid and experimental conditions may differ slightly from the theoretical conditions, so the exact concentration criteria are slightly different. However, the peak point of the output voltage is also present in the numerical model, and most predicted values are within a 20% error range.

## Conclusion

In this study, a novel environmentally friendly electrical energy harvester has been proposed using a mixture of glycerol and pure water. To examine the effects of the glycerol mixture, the dynamic behaviors of an oscillating liquid bridge between hydrophobic and hydrophilic surfaces were numerically modeled, and the generation of electrical power was calculated from the RC-circuit model with modulated EDLCs and integrated. The numerical results were compared with the experimental results using various concentrations of glycerol mixtures as working fluids. A peak point of the voltage output occurred at a specific region of the glycerol concentration in both the experimental and numerical results.

The total contact area difference decreased while the dielectric constant of the fluid increased with increasing concentration of the glycerol mixture. Theoretically, when the dielectric constant of the fluid decreases, the voltage output should increase, but the decrease of the contact-area difference results in lower total electrical power generation. This trade-off produces a peak value in the output and enhances the energy density by up to 2.23 times compared to that obtained using pure water. It is expected that higher harvesting efficiency can be achieved by modulating the physical properties of the working fluids of liquid bridges.
